# The 12 Gastrointestinal Pathogens Spectrum of Acute Infectious Diarrhea in a Sentinel Hospital, Shenzhen, China

**DOI:** 10.3389/fmicb.2016.01926

**Published:** 2016-11-30

**Authors:** Hongwei Shen, Jinjin Zhang, Yinghui Li, Sirou Xie, Yixiang Jiang, Yanjie Wu, Yuhui Ye, Hong Yang, Haolian Mo, Chaoman Situ, Qinghua Hu

**Affiliations:** ^1^Futian District Center for Disease Control and PreventionShenzhen, China; ^2^Shenzhen Major Infectious Disease Control Key Laboratory, Shenzhen Center for Disease Control and PreventionShenzhen, China; ^3^Peking University Shenzhen HospitalShenzhen, China

**Keywords:** gastrointestinal pathogen, spectrum, acute infectious diarrhea, sentinel hospital, Shenzhen

## Abstract

Acute infectious gastroenteritis is one of the most common diseases among all ages, particularly in developing countries. The pathogen spectrum may differ among different regions and seasons. To investigate the etiology of acute diarrhea in Shenzhen, a prospective study was conducted from August 2014 to September 2015. Stools from 412 patients with diarrhea (286 of whom were adults) including the general epidemiological information of the patients were collected. The 19 pathogens were detected by conventional culture method or multiplex PCR assay, which included five viruses (rotavirus, adenovirus, sapovirus, norovirus, and astrovirus), 11 bacterial pathogens (*Salmonella, Campylobacter jejuni, Shigella, Listeria monocytogenes, Vibrio parahaemolyticus, Vibrio cholera*, Enterohemorrhagic (EHEC), enteropathogenic (EPEC), enteroinvasive (EIEC), enterotoxigenic (ETEC); and enteroaggregative *Escherichia coli* (EAEC)) and three parasites (*Entamoeba histolytica, Giardia lamblia*, and *Cryptosporidium parvum*). A potential pathogen and coinfection was found in 41.5 and 7.0% of cases, respectively. The bacterial infection was the dominant cause of diarrhea (32.3%), and the three most frequently identified organisms were *Salmonella* (12.1%), ETEC (8.0%), and *Campylobacter jejuni* (4.9%). *Salmonella* enteritidis was the leading serotype of *Salmonella* sp. Norovirus (8.3%) and sapovirus (2.2%) were the most common viral pathogens, followed by adenovirus (1.5%) and rotavirus (1.2%). No EHEC, *L. monocytogenes, V. cholera, Shigella*, and parasites were found. The single most important causes of diarrhea were *Salmonella* spp. and *Campylobacter jejuni*, which points toward the need for testing and surveillance for these pathogens in this region.

## Introduction

Acute infectious diarrhea accounts for substantial morbidity and mortality in all regions in the world and among all ages (Fischer Walker et al., 2010), with over 2 million death occurring each year, particularly among infants younger than 5 years (Clarke et al., 2003; Kosek et al., 2003). In developing countries, such as China, the associated mortality is high because of the high incidence and poor medical condition in some regions. A broad range of organisms have been recognized as the etiologies of acute infectious diarrhea. Rotavirus is known as the most common cause of severe acute gastroenteritis among children (Parashar et al., 2003). Norovirus infection is responsible for most outbreaks of nonbacterial gastroenteritis (Yan et al., 2003). *Shigella* sp., *V. parahaemolyticus, Salmonella* sp., and diarrheagenic *Escherichiacoli* (DEC) are frequently detected agents associated with acute infectious diarrhea (Huilan et al., 1991; Bai et al., 2004; Qu et al., 2012). *C. jejuni* is one of the most common causes of gastroenteritis worldwide and its prevalence is particularly high in developed countries (Allos, 2001; Koopmans et al., 2001; Scallan et al., 2011). Furthermore, some less common pathogens, such as astrovirus, and parasites may also cause acute diarrhea (Olesen et al., 2005; Denno et al., 2012).

Although these common enteropathogens have been extensively studied in China (Bai et al., 2004; Qu et al., 2012; Li et al., 2014), and the laboratory-based surveillance for acute infectious diarrhea has been established in Shenzhen City since 2007, few comprehensive studies covering a broad range of diarrheal agents have been undertaken. Furthermore, the long-term fluctuation in the frequency of infections with the common gastrointestinal pathogens captured by this surveillance system suggested the etiology of acute infectious diarrhea in this region has changed obviously during the past few years. To investigate the etiology of diarrhea, thus informing policy makers on future vaccine and intervention development in the southern coastal region of China, the present study, comprising testing for bacteria, virus, and parasites, was conducted.

## Materials and Methods

### Specimen Collection

Acute diarrhea was defined as ≥3 passages of watery, loose, mucus-, or bloody-stools during a 24-h period. Stool samples were consecutively collected by Peking University Shenzhen Hospital from patients who presented with acute diarrhea to the diarrhea outpatient clinics during the period from August 2014 through September 2015. A total of 412 patients without receiving antibiotics treatment could provide a stool specimen during the visit, aged from 1 month to 78 years old, were enrolled in this study. A brief questionnaire including demographic data and clinical signs was filled out from patients or their guardians. The feces were stored at 4°C and transported to the laboratory of Futian District Center for Disease Control and Prevention (Futian CDC) in two days during workdays or 3 days during weekends.

### Sample Processing

The stool samples were enriched in selective broth medium or cultured on different medium immediately to obtain bacterial strains. If the stool sample volumes were insufficient, the stools were diluted using PBS before bacterial culture. The remaining specimens were prepared as 20% stool suspension in PBS and kept at -60°C until further study. The viral and bacterial nucleic acid was extracted and purified using the High Pure Viral Nucleic Acid Kit (Roche Diagnostics, Mannheim, Germany) and QIAamp Fast DNA Stool Mini Kit (Qiagen GmbH, Hilden, Germany), respectively, according to the manufacturer’s recommended protocols. The stool suspension was heated for 10 min at 100°C and then DNA of parasites was isolated with QIAamp DNA Mini Kit (Qiagen GmbH, Hilden, Germany). Purified nucleic acid was frozen at -80°C in 15-μl aliquots.

### Bacteria Isolation

The CHROM agars were used for isolation of *Salmonella* sp., *Vibrio parahaemolyticus*, and *Vibrio cholera*; HE medium and *Campylobacter*-selective agar for *Shigella* sp. and *C. jejuni*, respectively. Specimens were enriched in selenite cysteine (SC) broth and 3% NaCl peptone water before plated on CHROM agar for isolation of *Salmonella* sp. and *Vibrio* sp., respectively. The *Salmonella* isolates from CHROM agar were identified using the Sensititre GNID (TREK Diagnostic System, Cleveland, OH, USA) and serotyped with a commercial serotyping kit (S&A Company, S&A Reagent Lab, Bangkok, Thailand) according to the manufacturer’s instructions. All the *V. parahaemolyticus* isolates were identified using the Sensititre GNID (TREK Diagnostic System, Cleveland, OH, USA) and serotyped with a commercial serotyping kit (Denka Seiken, Tokyo, Japan), followed by real-time PCR detection of the thermostable direct hemolysin (*tdh*) and thermolabile hemolysin (*trh*) genes (Blackstone et al., 2003; Davis et al., 2004). Stool samples were examined for diarrhoeagenic *E. coli* by detecting virulence loci of a pool of three suspicious clonies from MacConkey Agar Plate, such as *eaeA, stx*_1_, and *stx*_2_ of enterohemorrhagic *E. coli* (EHEC); *eaeA* and *escV* of enteropathogenic *E. coli* (EPEC); *ipaH* of enteroinvasive *E. coli* (EIEC) and *Shigella* strains; *lt, sth*, and *stp* of enterotoxigenic *E. coli* (ETEC); and *aggR* of enteroaggregative *E. coli* (EAEC) (Chen et al., 2014). To reduce the risk of false negative results, the multiple PCR method simultaneously detecting five bacterial pathogens associated with diarrhea and the PCR assay targeting DEC were adopted to screening the purified nucleic acid (Chen et al., 2014; Hu et al., 2014).

The oligonucleotide primers and probes used for real-time PCR for simultaneous detection of virulence loci of DEC and simultaneous detection of five enteric bacteria were listed in **Table 1**. A common tag sequence was added at the 5′ end of each primer to minimum the formation of primer dimer. The amplification reaction was the same as previously described (Chen et al., 2014; Hu et al., 2014). The cycling conditions included a initiating stage of 3 min at 95°C followed by 5 cycles of 10 s at 95°C, 20 s at 58°C, and 15 s at 72°C and finally by a 40 cycles of 10 s at 94°C, 20 s at 55°C, and 15 s at 72°C (Bio-Rad CFX96, Bio-Rad, USA).

**Table 1:** The oligonucleotide primers and probes for real-time PCR used in this study.

Organism	Target gene^a^	Primer sequence (5′-3′)^b^	Probe (5′-3′)	Citation
Tag	/	GCAAGCCCTCACGTAGCGAA	/	Chen et al., 2014
**DEC**				
ETEC^1^	*stp*	F: AAAAGCGAGTGTACCTCGACA R: CAGTTGACTGACTAAAAGAGGGG	HEX-CGCGTCTCAAATATCCGTGA AACAACATGACGCG	Chen et al., 2014
ETEC^1^	*sth*	F: GTGGTCCTGAAAGCATGAATAG R: CAACAAAGCAACAGGTACATACG	FAM-CGCGGTGAATTGTGTTGTAATCCTGCTTG TACCGCG	Chen et al., 2014
ETEC^1^	*lt*	F: ACAGGAGGTTTCTGCGTTAG R: GGTGGGAAACCTGCTAATCT	ROX-CGCCGGTATTACAGAAATCTGAA TATAGCTCCGGCG	Chen et al., 2014
EAEC^1^	*aggR*	F: TGCAAAAGAAGAAATCAACAGT R: CAGAATCGTCAGCATCAGCTAC	CY5-CGGACAAAAGTAGATGCTTGCAGTTGTCCG	Chen et al., 2014
EIEC^2^	*ipaH*	F: GAAAACCCTCCTGGTCCATC R: GTCTGGAAGGCCAGGTAGACTT	FAM-CCCGGCTGGAGGACATTGCCCGGG	Chenet al., 2014
EPEC&EHEC^2^	*eaeA*	F: GTAACCAGGCTTCGTCACA R: AAGGAAAAAACGCTGACCCG	CY5-CCCAGTGGTAATAACTTTGACGGTAG TTCACTGGG	Chen et al., 2014
EPEC&EHEC^2^	*escV*	F: GGCTCTCTTCTTCTTTATGGCTG R: GGGAAAGAAGTTAGTTCAAGAGGAT	HEX-CCCGCGCAACAGTTGTGGTGGATATCATTAT CGCGGG	Chen et al., 2014
EHEC^2^	*stx1*	F: ASAGCGGTTACATTGTCTGGT R: CTGCGTCAGTGAGGTTCCA	ROX- CCGCGTACGGGGATGCAGA TAAATCGCGG	Chen et al., 2014
EHEC^2^	*stx2*	F: CATGACAACGGACAGCAGTTA R: TCTGGTCATTGTATTACCACTGAA	ROX-CCGCCACTCACTGGTTTCATCATATCTGGCGG	Chen et al., 2014
**Bacteria**				
*L. monocytogenes*^3^	*hly*	F: TGCAAGTCCTAAGACGCCA R: CACTGCATCTCCGTGGTATACTAA	ROX-CGCGCTTGTATATACTTATCGATTTC ATCCGCGCG	Hu et al., 2014
*V. parahaemolyticus*^3^	*toxR*	F: AAGCGCCAGTAGTACCTGA R: CCAATCTGACGGAACTGAGATT	FAM/HEX-CGGCAAATCGGTAGTAATAGTGCCG	Hu et al., 2014
*Salmonella*^3^	*SPI*	F: GAACCTGGCCTGAAGACATAAA R: AGGTCAATAGCCAGAAAGGGA	CY5-CCGGCTAACTGACTCACCGTAAATGCCGG	Hu et al., 2014
*C. jejuni*^3^	*gyrA*	F: AGTGCG(C/A)GCTAAAACTCA R: CAAGCTCTGCAATCTGCTC	FAM-CCGCCTTATCAAACCAATAAAGCTAGGCGG	Hu et al., 2014
*Shigella*^3^	*ipaH*	F: TGAAGGAAATGCGTTTCTATG R: AGGGAGAACCAGTCCGTAAA	HEX-CACGGCCGAAGCTATGGTC AGAAGCCGTG	Hu et al., 2014
**Virus**				
Sapovirus^4^	RdRp	F: CTCGCCACCTACAATGCYTGGTT R: TGGGATGTGGTCGGVCCAGT	FAM-CCGAGCCTAGTGTTTGAGATGGAGGGCAAT GGCTCGG	Jiang et al., 2014
Sapovirus^4^	VP1	F: ACRGCCAARGCTGAGGGG R: CCCTCCATTTCAAACACTAATT	FAM-CCCTGGGCCCCAGTGAAGAGACCACCAGGG	Jiang et al., 2014
Norovirus GI^4^	VP1	F: CGCTGGATGCGNTTCCAT R: CCTTAGACGCCATCATCATTTAC	FAM/HEX-CCGTGGAAGATYGCG RTCTCCTGTCCACGG	Jiang et al., 2014
Norovirus GII^4^	VP1	F: ATGTTCAGRTGGATGAGRTTCTCWGA R: TCGACGCCATCTTCATTCACA	ROX-CGCACGATCGCCCTCCCACGTGCG	Jiang et al., 2014
Adenovirus^4^	hexon	F: TACTTCAGCCTGGGGAACAAG R: CAGCGTAAAGCGCACTTTG	CY5-CCCACGCCTGTCTGTGGTT ACATCGTGGG	Jiang et al., 2014
Rotavirus^4^	NSP3	F: ACCATCTACACATGACCCTC R: CACATAACGCCCCTATAGCC	HEX-CCGAGCACAATAGTTAAAA GCTAACACTGTCGCTCGG	Jiang et al., 2014
Astrovirus^4^	ORF1	F: ATCCGTGATGTTAATGGG R: CGTTGCCAGAAAAGAAGC	FAM/CY5-CGCGACACCCCTGAA GGGAAAGGGACAGTCGCG	Jiang et al., 2014
**Parasites**				
*E. histolytica*^5^	SSU rRNA	F: ATTGTCGTGGCATCCTAACTCA R: GCGGACGGCTCATTATAACA	VIC-TCATTGAATGAATTGGCCATTT	Verweij et al., 2004
*G. lamblia*^5^	SSU rRNA	F: GACGGCTCAGGACAACGGTT R: TTGCCAGCGGTGTCCG	FAM-CCCGCGGCGGTCCCTGCTAG	Verweij et al., 2004
*C. parvum*^5^	DNAj-like	F: CGCTTCTCTAGCCTTTCATGA R: CTTCACGTGTGTTTGCCAAT	Texas Red –CCAATCACAGAATCA TCAGAATCGACTGGTATC	Verweij et al., 2004

*^a^*stp, sth*, heat-stable enterotoxin; *lt*, heat-labile enterotoxin; *aggR*, transcriptional regulator aggR; *ipaH*, invasive plasmid antigen H; *eaeA*, intimin; *escV*, enterocyte effacement gene locus; *stx1*, shiga toxin 1; *stx2*, shiga toxin 2; *hly*, listeriolysin O; *toxR*, transmembrane regulatory protein; *SPI*, specific pathogenicity island; *gyrA*, DNA gyrase subunit A; RdRp, RNA-dependant RNA Polymelase; VP1, capsid protein VP1; NSP3, nonstructural protein 3; ORF1, nonstructural protein; SSU rRNA, small subunit ribosomal RNA. ^b^F, forward; R, reverse. ^1^ tube 1; ^2^ tube 2; ^3^ tube 3; ^4^ tube 4; ^5^ tube 5.*

### Virus Detection

Reverse transcription-PCR (RT-PCR) was performed by using PrimerScript First strand cDNA Synthesis Kit (TaKaRa, China). Viral RNA was reverse transcribed for 10 min at 30°C, 50 min at 42°C, and 5 min at 95°C. A single real-time multiplex PCR assay was followed for simultaneous detection of five enteric viruses: norovirus genogroups I and II; sapovirus genogroups I, II, IV, and V; human rotavirus A; adenovirus serotypes 40 and 41; and human astrovirus (Jiang et al., 2014). The specific primers and probes used in this reaction were presented in **Table 1**. Each primer had a common tag sequence at the 5′ end of the sequence. A combination of two probes for Norovirus GI was designed in order to differentiate the five organisms under four detecting channels. The amplification reaction was consistent with previously described and cycling condition was the same as that of simultaneous detecting virulence loci of DEC or five enteric bacteria (Jiang et al., 2014).

### Parasites Detection

A multiplex PCR method with high sensitivity and specificity was adopted to simultaneous detection of the three most common diarrhea-causing parasitic protozoa, including *Entamoeba histolytica, Giardia lamblia*, and *Cryptosporidium parvum* (Verweij et al., 2004).

All primers and detection probes used for real-time PCR for simultaneous detection of the three parasites were described in **Table 1**. Amplification reactions were carried out as previously described (Verweij et al., 2004), which consisted of 15 min at 95°C followed by 40 cycles of 15 s at 95°C, 30 s at 60°C, and 30 s at 72°C. Amplification, detection and data analysis were performed in a Bio-Rad CFX96 real-time thermal cycler (Bio-Rad, USA).

### Ethics Statement

All aspects of the study were performed in accordance with national ethics regulations and approved by the Ethics Committee of Peking University Shenzhen Hospital, as well as Futian District CDC. Participants received information on the study’s purpose and of their right to keep information confidential. Written consent was obtained from each adult participant and children’s parents or their guardians.

## Results

### Epidemiological Information

A total of 412 cases (184 of whom were part of the transient population) were enrolled in the prospective study in the12-month period. Patients ranged in age from 1 month to 78 years (median 28 years). Of all the patients, 211 (51.2%) had abdominal pain, 68 (16.5%) had vomiting, 28 (6.8%) had fever, and 14 (3.4%) had blood in stools, respectively (**Table 2**). Stools from 297 of all cases contain enough material for thorough microbiologic evaluation without dilution. Overall, a potential pathogen was found in 41.5% of all cases who had loose or liquid stools.

**Table 2:** The epidemiological and clinical characteristics of samples (*n* = 412) in this study.

Category	Subcategory	No. (%)
Sex	Female	231 (56.1)
	Male	181 (43.9)
Age (years)	0~4	107 (26.0)
	5~9	5 (1.2)
	10~19	25 (6.1)
	20~29	71 (17.2)
	30~39	71 (17.2)
	40~49	61 (14.8)
	50~59	42 (10.2)
	60 and above	30 (7.3)
Residence	Transient population	228 (55.3)
	Local population	184 (44.7)
Clinical Manifestations	Abdominal pain	211 (51.2)
	Vomiting	68 (16.5)
	Fever	28 (6.8)
	Blood in stools	14 (3.4)

### Gastrointestinal Pathogens Spectrum

Among all samples tested, a bacterial pathogen was found in 107 (26.0%) of cases (*Salmonella* sp., 37 specimens; ETEC, 20; *C. jejuni*, 18; EPEC, 16; EAEC, 8; *V. parahaemolyticus*, 4; and EIEC, 4); a virus was found in 35 (8.5%) of cases (norovirus GII, 24 specimens; adenovirus, 4; sapovirus, 3; rotavirus, 3; and astrovirus, 1); both viral and bacterial infection was found in 15 (3.6%) of cases (EAEC and sapovirus, 2 specimens; ETEC and sapovirus, 2; ETEC and norovirus GII, 2; *C. jejuni*, and norovirus GII 2; EPEC and norovirus GII, 1; EPEC and adenovius, 1; *Salmonella* sp. and norovirus GII, 1; *Salmonella* sp. and sapovirus, 1; *Salmonella* sp., EPEC and norovirus GII, 1; *Salmonella* sp., ETEC and rotavirus, 1; *Salmonella* sp., and adenovirus and sapovirus, 1). Mixed bacterial infection was found in 11 (2.7%) of cases (*Salmonella* sp. and ETEC, 5 specimens; EPEC and ETEC, 2; *Salmonella* sp. and EPEC, 1; *Salmonella* sp. and EAEC, 1; EPEC and EAEC, 1; *Salmonella* sp., EPEC and ETEC, 1). Sample from one patient yielded norovirus GII and rotavirus; and from two patients yielded both norovirus GII and astrovirus. No EHEC, *L. monocytogenes, Shigella, V. cholera*, and parasites were isolated or found (**Table 3**).

**Table 3:** Pathogens detected in 412 tool samples isolated from the acute diarrhea patients by culture/ multiplex PCR method.

Organism	No. of patients	Co-infection
*C. jejuni*	20	2 with norovirus GII
EAEC	12	2 with sapovirus, 1 with *Salmonella*, and 1 with EPEC
EIEC	4	
EPEC	24	2 with ETEC, 1 with *Salmonella*, 1 with adenovirus, 1 with norovirus GII, 1 with *Salmonella* and ETEC, and 1 with *Salmonella* and norovirus GII
ETEC	33	5 with *Salmonella*, 2 with sapovirus, 2 with norovirus GII, and 1 with *Salmonella* and rotavirus
*Salmonella* sp.	50	1 with sapovirus, 1 with norovirus GII, and 1 with sapovirus and adenovirus
*V. parahaemolyticus*	4	
Adenovirus	6	
Norovirus	34	2 with astrovirus, 1 with rotavirus
Rotavirus	5	
Sapovirus	9	
Astrovirus	3	
EHEC	0	
*L. monocytogenes*	0	
*Shigella*	0	
*V. cholera*	0	
*E. histolytica*	0	
*G. lamblia*	0	
*C. parvum*	0	

The seasonal prevalence was most prominent for *Salmonella, V. parahaemolyticus*, ETEC, EAEC, and EPEC, with most infections seen in summer season (April through October), including 49 of 50 *Salmonella* infection, 4 of 4 *V. parahaemolyticus*, 32 of 33 ETEC, 11 of 12 EAEC, and 23 of 24 EPEC were found during this period. The infection with norovirus tended to occur throughout the year. The viral infection was commonly found in children aged under 5 years. *Salmonella* was seemly found in all age groups with high prevalence (**Table 4**).

**Table 4:** Age distribution of Shenzhen acute diarrhea patients caused by the most common pathogens^a^.

Age (yr)	All patients	No. (%)								
		*Salmonella* sp.	*C. jejuni*	*V. parahaemolyticus*	EAEC	EIEC	EPEC	ETEC	Adenovirus	Norovirus	Rotavirus	Sapovirus
0~4	107	17 (15.9)	4 (3.7)	1 (0.9)	6 (5.6)	0 (0)	6 (5.6)	3 (2.8)	3 (2.8)	13 (12.1)	1 (0.9)	5 (4.6)
5~9	5	1 (20.0)	1 (20.0)	0 (0)	0 (0)	0 (0)	1 (20.0)	0 (0)	0 (0)	0 (0)	0 (0)	0 (0)
10~19	25	4 (16.0)	2 (8.0)	0 (0)	0 (0)	0 (0)	1 (4.0)	1 (4.0)	0 (0)	1 (4.0)	0 (0)	1 (4.0)
20~29	71	8 (11.3)	5 (7.0)	1 (1.4)	1 (1.4)	3 (4.2)	2 (2.8)	5 (7.0)	1 (1.4)	5 (7.0)	0 (0)	1 (1.4)
30~39	71	7 (9.9)	3 (4.2)	2 (2.8)	0 (0)	0 (0)	2 (2.8)	7 (9.9)	2 (2.8)	8 (11.2)	1 (1.4)	0 (0)
40~49	61	6 (9.8)	2 (3.3)	0 (0)	4 (6.6)	1 (1.6)	7 (11.5)	5 (8.2)	0 (0)	0 (0)	1 (1.6)	1 (1.6)
50~59	42	5 (11.9)	0 (0)	0 (0)	1 (2.4)	0 (0)	3 (7.1)	8 (19.0)	0 (0)	4 (9.5)	2 (4.7)	1 (2.4)
60~78	30	2 (6.7)	3 (10.0)	0 (0)	0 (0)	0 (0)	2 (6.7)	4 (13.3)	0 (0)	3 (10.0)	0 (0)	0 (0)

*^a^Percentages are shown in parentheses. Mixed infections are included in this table.*

### Serotyping and Genotyping

One of the most frequently isolated bacterial pathogen was *Salmonella* sp. Several serotypes were identified: serovars Enteritidis, Senftenberg, Typhimurium, Litchfield, Stanley, London, Derby, Essen, Chomedy, Fillmore, Manchester, Papuana, Uganda, and untypable in 12, 5, 4, 4, 4, 2, 1, 1, 1, 1, 1, 1, 1, and 2 patients, respectively (**Table 5**). *C. jejuni* was identified from 20 cases, and was the third most common bacterial cause of diarrhea. All the three *V. parahaemolyticus* isolates of serotype O3:K6 were *tdh*+*trh*-. The suspected colonies from 47 samples were DEC positive, including 1 EIEC, 8 EAEC, 16 EPEC, and 22 ETEC identified by multiplex PCR assay, whereas additional 3 EIEC, 3 EAEC, 7 EPEC, and 9 ETEC were identified in purified nucleic acids from stool samples. Twenty of twenty-two EPEC infections were *eaeA* positive. Of the 33 ETEC infections, 14 were *lt* positive, 14 were *sth* positive, 4 were *stp* positive, and one were *sth*+*lt* positive. The isolation results of *V. cholera* and *Shigella* were in consistent with PCR method. Additional 10 *Salmonella* sp., and 1 *V. parahaemolyticus* were detected by PCR assay not culturing, whereas additional 7 *Salmonella* sp. strains were isolated by culture other than PCR assay.

**Table 5:** Distribution relative to patient age and gender of 40 *Salmonella enterica* isolates from acute diarrhea infections in Shenzhen, August 2014 through September, 2015.

Age (year)	No. of isolates	No. of		No. of isolates of					
		Males	Females	Serovar Enteritidis	Serovar Typhimurium	Serovar Senftenberg	Serovar Litchfield	Serovar Stanley	Remaining serotypes
0~4	13	8	5	3	3	0	1	1	5
5~9	1	1	0	1	0	0	0	0	0
10~19	3	2	1	2	0	0	0	1	0
20~29	6	3	3	2	0	2	0	1	1
30~39	7	0	7	3	0	1	2	0	1
40~49	5	0	5	0	1	1	1	1	1
50~59	5	1	4	1	0	1	0	0	3
60~78	0	0	0	0	0	0	0	0	0
Total	40	15	25	12	4	5	4	4	11

## Discussion

As the youngest city and one of the most vibrant cities in China, Shenzhen owned a special demographic feature. Young adults and transient population constituted a large percentage of the whole residents. As a result, the majority of the patients belonged to age group of 20~49 years old (203/412, 49.3%) and 55.3% of the patients lived in the city transiently. There was no difference for the frequency of infection observed between the age group 20~49 years old (41.6%) and others (41.4%). However, the percentage of infection was significantly higher among transient population (104/228, 45.6%) compared with the residents (67/184, 36.4%), which could be attributed to the residents’ better hygiene condition. The weather would be another factor for affecting the pathogen spectrum of the acute diarrhea. The summer season in Shenzhen could last from April through October and 372 of 412 samples were collected during this period. The prevalence of infection during April and October (159/372, 42.7%) was higher than that during other months (12/40, 30.0%). This might due to the hot weather and rainy months which were associated with some bacterial infections (Adkins et al., 1987; Carlton et al., 2013).

A potential pathogen was detected for 41.5% of patients with acute diarrhea in the present study and coinfection was found for 7.0% of cases. The bacterial infection was the dominant cause of acute diarrhea in the southern coastal region of China, surpassing viral and parasite infection. DEC, *Salmonella* sp., and *C. jejuni* were the most frequently identified pathogens, followed by noro-, sapo-, adenovirus, rotavirus, and *V. parahaemolyticus*. *C. jejuni* has generally not been tested for in microbiology laboratories in China, and the incidence of *C. jejuni* is therefore been seriously underestimated.

A number of case-control studies and cohorts on diarrhea have been conducted in the Netherlands (De Wit et al., 2001; Koopmans et al., 2001), the United Kingdom (Tam et al., 2012), and Australia (Barnes et al., 1998), and in these studies viruses were found to be the leading cause of diarrhea in community cases. Norovirus was observed as the most frequent pathogen in all age groups in the Dutch study (De Wit et al., 2001). Norovirus was the most commonly identified viral pathogen with a prevalence of 8.3% in this study. The associated infection could be found in all age groups except for age group 5~9 years and 40~49 years. This may due to the small sample size, as no association was found between the patient age and norovirus prevalence (Ahmed et al., 2014). The percentage attributable to noro-, sapo-, and rotavirus was clearly higher than our study (Koopmans et al., 2001). The viruses caused milder symptoms than bacterial pathogens, and thus patients with viral infections would be less likely to seek medical attention (Saphra and Winter, 1957; Lopman et al., 2004; Chen et al., 2007). Community studies are therefore expected to disclose a higher proportion of viral infections than studies involving patients admitted to hospitals. The fact that our specimens were collected clustering in April through November (August, 2014 to November, 2014 and April, 2015 to September, 2015) may also diminished the frequency of viruses’ detection, because the seasonal prevalence for viruses was seen in the period from January to May (Olesen et al., 2005).

Diarrheagenic *Escherichiacoli* was found in 16.5% of patients with diarrhea and identified as the most common enteric pathogen in the present study. Of the 68 DEC infections, 45 were identified as positive by both isolation and direct PCR assay, and 3 and 20 were identified as positive by only isolation or direct PCR assay, respectively. The direct PCR method was much more sensitive than the conventional culture, which may due to the errors of selecting putative colonies. A total of 29.4% of cases with DEC infection were co-infected with other organisms, showing the weak correlation between DEC infections with diarrhea. Some notable features occurred in DEC infections, including that most EAEC infections (7/11) were found in May and June, and ETEC infections (33/34) were mainly found in hot wet months of May through September and one ETEC strain isolated from a male patient aged 44 years harbored both *sth* and *lt* virulence gene, both *sth* and *lt* positive was not found frequently. These features needed a larger sample size to be confirmed and may be predictive for clinical diagnostics.

Of the13 serotypes of *Salmonella* sp. identified in this study, the serovar Enteritidis was dominant throughout the surveillance period, followed by *S*. Senftenberg, *S*. Typhimurium, *S*. Litchfield, and *S*. Stanley. In addition to these *Salmonella* serotypes, some less common serotypes were identified, which included *S.* Uganda, *S.* Chomodey, *S.* Fillmore, and *S.* papuana. A total of two isolates were untypable. With regard to *S*. Enteritidis, our finding was in agreement with previous study conducted in Guangdong Province that *S*. Enteritidis mainly caused human salmonellosis in adults (Ke et al., 2014). However, the number of patients caused by *S*. Senftenberg ranked the second, which differed from previous study conducted in this area but was consistent with the surveillance data in Shanghai, China (Xu et al., 2009). The Shanghai study showed that *S*. Senftenberg was the second most common identified *Salmonella* serotype in individuals in foods and service industries (Zhu et al., 2008), and thus the customers were more likely infected with *S*. Senftenberg through contaminated foods, if hygiene and cooking practices were not adequate. More interestingly, among five strains of *S*. Senftenberg recovered from diarrheal patients, two atypical strains’ hydrogen sulfide was negative. The phenotype and genetic character of these atypical *Salmonella* strains is under way.

*Campylobacter jejuni* infections has been the leading cause of bacterial gastroenteritis reported in the developed countries (Tauxe et al., 1992; Altekruse et al., 1999). However, few surveillance of *C. jejuni* was conducted and the associated disease burden was seriously underestimated in China. In the present study, 4.9% (20/412) of samples was found to be positive for *C. jejuni*, indicating that *C. jejuni* has been an important pathogen in Shenzhen. The detection rate was significantly higher than that reported in other cities of China including Yangzhou, Beijing, and Wuxi (Zhou et al., 2008; Huang et al., 2009; Liu et al., 2014), which may due to the common consumption of chicken and meat in southern China. *C. jejuni* was found in many foods of animal origin. Surveys of raw agricultural products support epidemiologic evidence implicating poultry, meat, and raw milk as sources of human infection (Altekruse et al., 1999). Most retail chicken and meat products were contaminated with *C. jejuni* (Boer and Hahné, 1990; Yang et al., 2003; ZhiFa et al., 2009), and thus the customers could easily get infections through eating undercooked or cross-contaminated foods. There were only two cases of coinfection found in *C. jejuni* infection, indicating the strong association with disease. In addition, An average number of five stools per day was found in *C. jejuni* infected patients, suggesting *C. jejuni* infection could cause severe diarrhea. The Yangzhou survey showed that children under 7 years of age had higher prevalence rate than that of other age groups (Xu et al., 2008), whereas the detection frequency was much higher in both age group 5~9 years (20.0%) and 60~78 years (10.0%) than that of children under 5 years old (3.7%) in this study. The discordance between the two surveys might be the result of regional differences and small sample size of the present study.

Only four cases of *V. parahaemolyticus* were detected, which differed from previous survey that *V. parahaemolyticus* has been the leading cause of bacterial infectious diarrhea in coastal regions of China (Li et al., 2014). The low frequency of isolation of *V. parahaemolyticus* may due to the improvement in hygiene and thus the consumption of undercooked or cross-contaminated shellfish decreased, which was commonly associated with *V. parahaemolyticus* infection. All the three isolates serotyped as O3:K6 were *tdh*+*trh*-, which was consistent with the surveillance data collected in Shenzhen that O3:K6 serotype was dominant in this region (Li et al., 2014).

The intestinal parasites infection was associated with the source of water supply, age and socioeconomic status (Leach et al., 2000). In the counties with poor sanitation, the prevalence of the three organisms could be very high (Kelsall and Ravdin, 1994; Leach et al., 2000). In addition, parasites*-*caused diarrhea could be commonly found in the immune-compromised individuals (Esfandiari et al., 1997). The improvement of sanitation in the big city, the consumption of boiled water, and no HIV-infected cases in these enrolled patients might contribute to the non-infection of intestinal parasites. Furthermore, the ability of PCR method in distinguishing *E. histolytica* and *E. dispar* could result in a lower but true prevalence of *E. histolytica* (Verweij et al., 2004), as more than 90% of *E. histolytica* infections are responsible for *E. dispar* (Clark, 1998). Similarly, the non-infection of EHEC may benefit from the habit of consumption of cooked food and improvement of hygiene, as most of the EHEC-associated outbreaks was transmitted through contaminated foods (Viazis and Diez-Gonzalez, 2011). It was consistent with our previous surveillance data that EHEC had a very low prevalence (4/11860, 0.03%) in this region (Chen et al., 2014).

This study had several limitations. Firstly, all the samples were collected in a hospital and the results may not be representative of distinct districts of this region. The hospital that diarrheal patients visited may be associated with their income, living address, and severity of illness which subsequently influence the prevalence of enteropathogens. Secondly, the limited sample size may influence the analysis of the seasonal patterns and population distribution of major enteropathogens. Therefore, the positive percentage of enteropathogens among some age groups may be overly represented due to the small sample size. Thirdly, only one-year-period data were collected, so long-term trends were not present in this study. Nevertheless, this was the most comprehensive data collection on the etiology of diarrhea in this region. The full range detection of entropathogens and the fact that the municipal sentinel hospital received patients from each district of Shenzhen made these results reasonably representative. It is anticipated that further long-term continuation and collection of surveillance data from more sentinel hospitals will be able to overcome these limitations.

## Conclusion

Bacterial pathogens were confirmed as the leading cause of acute infectious diarrhea in outpatients in Shenzhen. DEC was the most common enteric pathogen and ETEC was mainly detected in hot wet months of May through September. The distribution of 13 serotypes identified revealed the diversity of *Salmonella* sp., which might explain the continuing epidemic of *Salmonella* sp. Infections in this region. The high frequency of single infection of *C. jejuni* and its associated severe manifestations indicate the strong association with diarrhea, and the disease burden attributed to *C. jejuni* has been seriously underestimated. The phenotype and genetic character of atypical *Salmonella* strains and a large surveillance survey on *C. jejuni* is under way. A long-term surveillance at more sentinel hospitals could provide a better understanding of diarrheal illnesses.

## Author Contributions

HS, JZ, and YL were involved in the collection of samples and detection of enteropathogens. SX and YJ collected the clinical data. YW, YY, and HY offered great help in the sample collection. HM and CS offered great help in the data analysis. QH designed this study. HS and QH drafted and revised this manuscript.

## Conflict of Interest Statement

The authors declare that the research was conducted in the absence of any commercial or financial relationships that could be construed as a potential conflict of interest.
